# Efflux-mediated resistance identified among norfloxacin resistant clinical strains of group B *Streptococcus* from South Korea

**DOI:** 10.4178/epih/e2014022

**Published:** 2014-10-11

**Authors:** Trang Nguyen Doan Dang, Usha Srinivasan, Zachary Britt, Carl F. Marrs, Lixin Zhang, Moran Ki, Betsy Foxman

**Affiliations:** 1 Department of Epidemiology, School of Public Health, University of Michigan, Ann Arbor, MI, USA; 2 Department of Cancer Control and Policy, Graduated School of Cancer Science and Policy, National Cancer Center, Goyang, Korea

**Keywords:** Fluoroquinolones, Norfloxacin, Ciprofloxacin, Levofloxacin, Moxifloxacin, Mutations, Efflux, Minimum inhibitory concentration

## Abstract

**OBJECTIVES:**

Group B *Streptococcus* (GBS), a common bowel commensal, is a major cause of neonatal sepsis and an emerging cause of infection in immune-compromised adult populations. Fluoroquinolones are used to treat GBS infections in those allergic to beta-lactams, but GBS are increasingly resistant to fluoroquinolones. Fluoroquinolone resistance has been previously attributed to quinolone resistance determining regions (QRDRs) mutations. We demonstrate that some of fluoroquinolone resistance is due to efflux-mediated resistance.

**METHODS:**

We tested 20 GBS strains resistant only to norfloxacin with no mutations in the QRDRs, for the efflux phenotype using norfloxacin and ethidium bromide as substrates in the presence of the efflux inhibitor reserpine. Also tested were 68 GBS strains resistant only to norfloxacin not screened for QRDRs, and 58 GBS strains resistant to ciprofloxacin, levofloxacin or moxifloxacin. Isolates were randomly selected from 221 pregnant women (35-37 weeks of gestation) asymptomatically carrying GBS, and 838 patients with GBS infection identified in South Korea between 2006 and 2008. The VITEK II automatic system (Biomerieux, Durham, NC, USA) was used to determine fluoroquinolone resistance.

**RESULTS:**

The reserpine associated efflux phenotype was found in more than half of GBS strains resistant only to norfloxacin with no QRDR mutations, and half where QRDR mutations were unknown. No evidence of the efflux phenotype was detected in GBS strains that were resistant to moxifloxacin or levofloxacin or both. The reserpine sensitive efflux phenotype resulted in moderate increases in norfloxacin minimum inhibitory concentration (average=3.6 fold, range=>1-16 fold).

**CONCLUSIONS:**

A substantial portion of GBS strains resistant to norfloxacin have an efflux phenotype.

## INTRODUCTION

Group B *Streptococcus* (GBS, *Streptococcus agalactiae*) is a major cause of neonatal sepsis and an emerging cause of infection in immune-compromised adult populations [1]. It is also a common member of the bowel microbiota. Both commensal and infecting GBS strains are often resistant to macrolides and fluoroquinolones, which are second-line GBS therapies [2-5].

Mutations in the quinolone resistance-determining region (QRDR) of the *gyr*Ase and topoisomerase genes are the dominant mechanism of fluoroquinolone resistance in GBS. We previously observed a high prevalence of QRDR mutations in GBS strains resistant to a range of fluoroquinolones, including norfloxacin, ciprofloxacin, levofloxacin, and moxifloxacin, among a large collection of commensal and invasive GBS isolates from South Korea. However, there were fluoroquinolone-resistant strains in our collection that did not harbor QRDR mutations; these strains were resistant only to norfloxacin [6].

Mutations affecting efflux pumps are a possible alternative mechanism for fluoroquinone resistance. Efflux pumps resulting in fluoroquinolone resistance have been previously reported in gram-positive organisms including *Staphylococcus aureus* [7-9], *Streptococcus pneumonia* [10-13], *Streptococcus pyogenes* [14] and Streptococcus suis [14]. A mutation affecting an efflux pump can cause antibiotic resistance either by increasing expression of the efflux pump following exposure to an antibiotic, or as a result of an amino acid substitution making the efflux pump more efficient at transporting the antibiotic. One antibiotic may be the substrate of one or more efflux pumps from the same or different efflux families [14,15].

We screened invasive and colonizing norfloxacin-resistant GBS isolates from South Korea without mutations in the QRDR regions of *gyr*Ase and topoisomerase genes for the efflux phenotype, using the efflux inhibitor method, in order to evaluate the possibility that efflux pump mutations are a mechanism of resistance to norfloxacin [13].

## MATERIALS AND METHODS

### Study collection

The sample collection and GBS isolation have been described previously [16]. Briefly, clinical isolates (identified as GBS isolated from urine, vagina, wounds, abscesses, cervix, prostate, blood, and sputum) were collected from hospitalized patients throughout South Korea, and colonizing isolates were specifically collected for this study from the urine, vagina, and rectum of healthy pregnant women receiving prenatal care at four hospitals in South Korea (Eulji Hospitals in Seoul and Daejeon, Cheil Hospital in Seoul, and Motae Women’s Hospital in Daejeon) between January 2006 and December 2008. Written informed consent was obtained from all participants, and the study protocol was approved by the institutional review board of Eulji University Hospital (04-08 and 06-25), and Cheil Hospital (SCH-IRB-2005-24). The use of these isolates for molecular studies was deemed exempt by the institutional review board at the University of Michigan (H03-00002617-R) because the Michigan investigators had no access to identifying information.

Susceptibility to quinolones was tested using a VITEK II (Biomerieux, Durham, NC, USA) at the Seoul Clinical Laboratories and Seoul Medical Science Institute with minimum inhibitory concentration (MIC) levels set according to the European Committee on Antimicrobial Susceptibility Testing (EUCAST) 2009 and 2011 guidelines [17]. Among the 1,075 GBS strains collected, 9.3, 9.5, and 0.8% were resistant to ciprofloxacin, levofloxacin, and moxifloxacin, respectively, and 95% were resistant to norfloxacin. Of the strains resistant to norfloxacin, 87% (886 strains) were sensitive to the other fluoroquinolones tested.

For the current study, we selected the 88 strains that were resistant only to norfloxacin. Twenty had been previously screened for QRDR mutations; no mutations in the QRDRs of the *gyr*Ase and topoisomerase genes were found. We therefore concluded that QRDR mutations were not the cause of this phenotype [6]. To increase our ability to detect the effect of a possible efflux pump mutation, we included 68 additional strains resistant only to norfloxacin that had not been screened for QRDR mutations. For comparison, we also included 56 strains that were resistant to ciprofloxacin, levofloxacin, or moxifloxacin in addition to norfloxacin; all had QRDR mutations, and two strains susceptible to all four fluoroquinolones were included as negative controls (Table 1). The 146 GBS strains tested for the efflux phenotype were classified into four different categories: i) no mutations in *gyr*A and *par*C, ii) mutations in *par*C only, iii) mutations in both *gyr*A and *par*C, and iv) mutations unknown (Table 1). Among the selected strains, 104 strains (71%) caused clinical disease and the remaining 42 strains (29%) were colonizing.

### Identification of efflux phenotype

We screened for a change in the MIC of norfloxacin in the presence of reserpine (20 μg/mL), an efflux pump inhibitor [18] to establish the efflux phenotype; all experiments were performed in duplicate. As a confirmation, we repeated the experiments using ethidium bromide as the substrate instead of norfloxacin [7,18,19]. GBS was grown in a Todd-Hewitt broth (Becton Dickinson, Sparks, MD, USA) at 37°C for 18 hours. A 0.5 McFarland standard suspension was prepared and transferred into a 96-well plate of Todd-Hewitt medium in the presence of different concentrations of norfloxacin or ethidium bromide. The final bacterial concentration in each well was -5×10^5^ colony forming units per milliliter. The plate was incubated at 37°C and MIC values were determined after 18 hours of incubation [20]. Norfloxacin was obtained from Sigma Aldrich (St Louis, MO, USA); reserpine was obtained from MP Biomedicals (Solon, OH, USA) and ethidium bromide was obtained from Fisher Scientific (Piscataway, NJ, USA). The MICs of two negative controls, GBS strains ATCC 12403 and A909, were both 4 μg/mL when either norfloxacin or ethidium bromide were used as a substrate; no difference in ethidium bromide MIC was detected for these control strains when reserpine was added. We used *Staphylococcus aureus* strain 1199B cloned with the norA efflux pump (provided by Dr. Glenn W. Kaatz, Wayne State University) as a positive control and GBS strains ATCC 12403 and A909 as negative controls.

We used the presence of a 4-fold difference in MIC for norfloxacin or ethidium bromide grown in the presence of reserpine compared to the absence of reserpine as a cutoff indicating the presence of the efflux phenotype. This cutoff is consistent with previous studies on *Streptococcus pyogenes* [21], *S. pneumonia, E. faecalis*, and *S. aureus* [10].

### Data analysis

All data analyses were performed using SAS version 9.2 (SAS Institute Inc., Cary, NC, USA).

## RESULTS

We included in our testing two GBS isolates that were sensitive to all fluoroquinolones tested, and nine that were insensitive to norfloxacin, but sensitive to the other fluoroquinolones. Of the 135 GBS strains resistant to norfloxacin alone or to norfloxacin and combinations of ciprofloxacin, levofloxacin, or moxifloxacin, 20 had no mutations in *gyr*A and *par*C,17 had mutations in *par*C only, 30 had mutations in both *gyr*A and *par*C, and the mutation status of 68 strains was unknown (Table 1).

Using norfloxacin as the substrate, half of the 20 strains resistant to norfloxacin with no QRDR mutations and 19% of those resistant to norfloxacin with a *par*C mutation showed evidence of the efflux phenotype (Table 2). The results for the 68 isolates resistant to norfloxacin with unknown mutations were essentially the same as that found for those without QRDR mutations: 52.9% showed evidence of efflux (the details of the mutations and efflux phenotype are presented in the Appendix 1). We found no difference in the prevalence of the efflux phenotype using norfloxacin as a substrate between clinical and colonizing isolates (p>0.05) or by site of isolation (p>0.05) (data not shown). For the two isolates sensitive to all fluoroquinolones and the 30 isolates resistant to norfloxacin with *par*C and *gyr*A mutations, there was no evidence of the efflux phenotype when grown in the presence of reserpine.

By contrast, when ethidium bromide was used as a substrate on the 88 strains resistant only to norfloxacin, we found only modest evidence for the efflux phenotype (29.4%). The results were the same for those without QRDR mutations and where QRDR mutations were unknown: 30% and 29%, respectively. A comparison of the results of the two detection methods, even when making the ethidium bromide test more sensitive by using a cutoff of a twofold change in MIC, found only moderate agreement between the two tests (kappa=0.6); 38 out of the 88 strains were efflux-positive using either ethidium bromide or norfloxacin as substrate while 9 strains were identified as efflux-positive only when using ethidium bromide and 8 strains were identified as efflux-positive only when using norfloxacin as a substrate.

## DISCUSSION

We identified an efflux phenotype among fluoroquinolone-resistant GBS strains without any QRDR mutations, detecting a 4-fold difference in norfloxacin MIC when grown the presence of reserpine. Strains with the efflux phenotype were resistant to norfloxacin but not ciprofloxacin, levofloxacin, or moxifloxacin. To our knowledge, this is the first report of efflux-mediated resistance to fluoroquinolones among colonizing and clinical strains of GBS. Efflux-mediated resistance to other antibiotics (macrolides [22,23], tetracycline [24]) in GBS has been previously reported.

We did not find the reserpine-mediated efflux phenotype in GBS strains resistant to fluoroquinolones when both *gyr*A and *par*C mutations were present. However, the efflux phenotype was found in three norfloxacin-resistant and ciprofloxacin intermediate-resistant strains with *par*C mutations but without *gyr*A mutations. This result is consistent with the possibility that efflux is the first step of low-level resistance to hydrophilic compounds like norfloxacin and ciprofloxacin [7,25]. Hydrophobic fluoroquinolones such as moxifloxacin and levofloxacin are thought to be poor substrates for efflux pumps in *Streptococcus pneumonia* [26] and *Staphylococcus aureus* [7,27], although reserpine-mediated efflux phenotypes have been reported for both these organisms [7,26,28]. Target alteration (i.e., mutations in *gyr*A and *par*C) accounts for higher levels of resistance, as has been reported in other Gram-positive bacteria [7,25]. Nonetheless, the presence of efflux-mediated resistance is concerning, as efflux pumps can be adapted to other substrates.

Among the 42 of 88 norfloxacin-resistant strains tested that did not show the efflux phenotype using norfloxacin as a substrate, nine were efflux-positive using ethidium bromide as a substrate. This suggests that multiple efflux pumps in GBS may coexist with varying substrate specificity. The presence of multiple efflux pumps has been demonstrated in other Gram-positive organisms, such as *S. aureus and S. pneumonia* [9,29]. It is therefore likely that our results on the prevalence of the efflux pump phenotype in GBS underestimate the true proportion of efflux-positive fluoroquinolone-resistant strains. Other methods of efflux detection, such as monitoring the gene expression of efflux pumps as has been done for *S. aureus*, will likely help to identify more efflux-positive strains. However, methods for gene expression based on quantitative polymerase chain reaction methodologies require that the genes involved in fluoroquinolone efflux be identified, which is not yet the case for GBS.

In summary, our study suggests the presence of an efflux phenotype in a substantial proportion of GBS strains resistant to norfloxacin. The reserpine-sensitive efflux phenotype resulted in moderate increases in the MIC of norfloxacin (average, 3.6-fold; range, >1-16-fold). We did not find the efflux phenotype in strains that were also resistant to other fluoroquinolones, such as ciprofloxacin, levofloxacin, and moxifloxacin, but did find it among strains with the combination of intermediate ciprofloxacin resistance and norfloxacin resistance. Future studies are needed to identify and characterize the mechanism underlying this phenotype as well as the genes causing fluoroquinolone efflux pump(s) in GBS.

## Figures and Tables

**Table 1.** Fluoroquinolone resistance of selected group B *Streptococcus* screened for the efflux phenotype. The 146 isolates were selected from 1,075 clinical and colonizing clinical group B *Streptococcus* isolates from South Korea (2006-2008)^1^

Norfloxacin	Ciprofloxacin	Levofloxacin	Moxifloxacin	Number of strains tested for efflux	QRDR mutations identified
S	S	S	S	2	No mutation
I	S	S	S	9	
R	S	S	S	20	
R	I	S	S	8	Mutations in *par*C only
R	I	I	S	8	
R	R	S	R	1	
R	R	I	S	6	Mutations in both *gyr*A and *par*C
R	R	R	S	13	
R	R	R	I	2	
R	R	R	R	9	
R	S	S	S	68	Mutations unknown
Total				146	

QRDR, quinolone resistance-determining region; S, susceptible; I, insensitive; R, resistant.

1 From Ki M, et al. Eur J Clin Microbiol Infect Dis 2012;31:3199-3205 [6].

**Table 2.** Minimum inhibitory concentration (MIC) of norfloxacin in the presence/absence of reserpine of 146 clinical group B *Streptococcus* strains from South Korea (2006-2008)^1^

Category	Count	Mean of MIC (μg/mL)		Average (range) of fold reduction in MIC between -/+ reserpine	% detected with reduction in MIC between -/+ reserpine
		- Reserpine	+ Reserpine		≥ 4 fold difference
No mutations					
Susceptible	2	4	4	1.0 (1-1)	0.0
Intermediate resistance to norfloxacin	9	13.3	7.6	2.2 (1-4)	33.3
Resistant to norfloxacin	20	44.8	14.2	4.0 (1-16)	50.0
Mutations in *par*C	17	41	31.5	1.6 (1-4)	18.8
Mutations in both *gyr*A and *par*C	30	128	128	1.0 (1-1)	0.0
Mutations unknown					
Resistant to norfloxacin	68	31.1	11.6	3.7 (1-16)	52.9

1 Norfloxacin MICs of two negative controls, group B *Streptococcus* strains ATCC 12403 and A909, were both 4 μg/mL; no difference in norfloxacin MICs was found when reserpine was added.

## Appendix 1. Association of efflux phenotype with presence of QRDR mutations among resistant and intermediate resistant group B *Streptococcus* strains isolated from South Korea

**Table:**

Susceptibility group and number of isolates^1^	Evidence of efflux phenotype^2^	QRDR mutations per group	Drug	Number of isolates per MIC (mg/L)^3^									
				1	2	4	8	16	32	64	128	256	>256
Group 1	No efflux	None detected	Norfloxacin			2							
(Susceptible group) (SSSS) (n=2)			Ciprofloxacin	2									
			Levofloxacin	2									
			Moxifloxacin	2									
Group 2	No efflux	None detected	Norfloxacin			3	3						
(ISSS) (n=6)			Ciprofloxacin	6									
			Levofloxacin	6									
			Moxifloxacin	6									
Group 3	With efflux	None detected	Norfloxacin				3						
ISSS (n = 3)			Ciprofloxacin	3									
			Levofloxacin	3									
			Moxifloxacin	3									
Group 4	No efflux	None detected	Norfloxacin				9	1					
RSSS (n = 10)			Ciprofloxacin	10									
			Levofloxacin	10									
			Moxifloxacin	10									
Group 5	With efflux	None detected	Norfloxacin				1	2	4	3			
RSSS (n = 10)			Ciprofloxacin	10									
			Levofloxacin	10									
			Moxifloxacin	10									
Group 6	With efflux	*par*C: 79 Ser-- > Phe	Norfloxacin		3				3				
RISS (n = 3)			Ciprofloxacin										
			Levofloxacin	3									
			Moxifloxacin	3									
Group 7	No efflux	*par*C: 79 Ser-- > Phe	Norfloxacin		5		2	1	2				
RISS (n = 5)			Ciprofloxacin										
			Levofloxacin	5									
			Moxifloxacin	5									
Group 8	No efflux	*par*C: 79 Ser-- > Phe	Norfloxacin				3	3	2				
RIIS (n = 8)			Ciprofloxacin		8								
			Levofloxacin Moxifloxacin	8	8								
Group 9	No efflux	*par*C: 79 Ser-- > Phe	Norfloxacin							5			
RRIS (n = 5)			Ciprofloxacin			5							
			Levofloxacin Moxifloxacin	5	5								
Group 10	No efflux	*par*C	Norfloxacin							1			
RRIS (n = 1)		79 Ser--> Phe	Ciprofloxacin			1							
		79 Ser-- > Tyr	Levofloxacin		1								
		83 Asp-->Gly	Moxifloxacin	1									
		*gyr*A:											
		81 Ser-- > Leu											
Group 11	No efflux	*par*C	Norfloxacin							14			
RRRS (n = 14)		79 Ser--> Phe	Ciprofloxacin			14							
		79 Ser--> Tyr	Levofloxacin			14							
		83 Asp-->Gly	Moxifloxacin	14									
		*gyr*A											
		81 Ser-- > Leu											
Group 12	No efflux	*par*C	Norfloxacin							2			
RRRI (n = 2)		79 Ser--> Phe	Ciprofloxacin			2							
		79 Ser--> Tyr	Levofloxacin			2							
		83 Asp-->Gly	Moxifloxacin		2								
		*gyr*A											
		81 Ser-- > Leu											
		85 Glu-->Lys											
Group 13	No efflux	*par*C	Norfloxacin								9		
RRRR (n=9)		79 Ser--> Phe	Ciprofloxacin				9						
		79 Ser--> Tyr	Levofloxacin			9							
		83 Asp-->Gly	Moxifloxacin			9							
		*gyr*A											
		81 Ser-- > Leu											

QRDR, quinolone resistance-determining region; MIC, minimum inhibitory concentration; R, resistant; S, susceptible; I, intermediate.

1 Order of fluoroquinolones is norfloxacin_ciprofloxacin_levofloxacin_moxifloxacin, eg. RSSS=Nor resistant and susceptible to ciprofloxacin, levofloxacin and moxifloxacin 68 strains resistant to only norfloxacin (RSSS) which had not been screened for QRDR mutations were also tested for efflux. Out of these 68 strains, 32 were efflux-negative and 36 were efflux positive.

2 Efflux phenotype is considered when there is at least four fold difference in norfloxacin MIC in the presence of reserpine.

3 MIC without reserpine: according to European Committee on Antimicrobial Susceptibility Testing (EUCAST) 2009 guidelines, norfloxacin and ciprofloxacin resistance cutoffs are at MIC>=4 mg/L; MIC breakpoint >=8 is for levofloxacin resistance and MIC>=4 for moxifloxacin resistant strains. From the more recent EUCAST 2011 guidelines, levofloxacin resistance is at MIC>2 and moxifloxacin resistant is at MIC>1 mg/L. Norfloxacin and ciprofloxacin breakpoints are not released as susceptibility testing to these drugs is not recommended due to GBS being poor target, for norfloxacin and ciprofloxacin the EUCAST 2009 guidelines are used of MIC cutoffs. Beige shaded boxes indicate MIC cutoff to indicate resistance to each fluoroquinolone , pink shaded box indicates intermediate resistance to ciprofloxacin.

## References

[1] Gibbs RS, Schrag S, Schuchat A (2004). Perinatal infections due to group B streptococci. Obstet Gynecol.

[2] Gonzalez JJ, Andreu A, Spanish Group for the Study of Perinatal Infection from the Spanish Society for Clinical Microbiology and Infectious Diseases (2005). Multicenter study of the mechanisms of resistance and clonal relationships of Streptococcus agalactiae isolates resistant to macrolides, lincosamides, and ketolides in Spain. Antimicrob Agents Chemother.

[3] Tazi A, Gueudet T, Varon E, Gilly L, Trieu-Cuot P, Poyart C (2008). Fluoroquinolone-resistant group B streptococci in acute exacerbation of chronic bronchitis. Emerg Infect Dis.

[4] Savoia D, Gottimer C, Crocilla’ C, Zucca M (2008). Streptococcus agalactiae in pregnant women: phenotypic and genotypic characters. J Infect.

[5] Wu HM, Janapatla RP, Ho YR, Hung KH, Wu CW, Yan JJ (2008). Emergence of fluoroquinolone resistance in group B streptococcal isolates in Taiwan. Antimicrob Agents Chemother.

[6] Ki M, Srinivasan U, Oh KY, Kim MY, Shin JH, Hong HL (2012). Emerging fluoroquinolone resistance in Streptococcus agalactiae in South Korea. Eur J Clin Microbiol Infect Dis.

[7] Kaatz GW, Seo SM, Ruble CA (1993). Efflux-mediated fluoroquinolone resistance in Staphylococcus aureus. Antimicrob Agents Chemother.

[8] DeMarco CE, Cushing LA, Frempong-Manso E, Seo SM, Jaravaza TA, Kaatz GW (2007). Efflux-related resistance to norfloxacin, dyes, and biocides in bloodstream isolates of Staphylococcus aureus. Antimicrob Agents Chemother.

[9] Kaatz GW, McAleese F, Seo SM (2005). Multidrug resistance in Staphylococcus aureus due to overexpression of a novel multidrug and toxin extrusion (MATE) transport protein. Antimicrob Agents Chemother.

[10] Zeller V, Janoir C, Kitzis MD, Gutmann L, Moreau NJ (1997). Active efflux as a mechanism of resistance to ciprofloxacin in Streptococcus pneumoniae. Antimicrob Agents Chemother.

[11] Jumbe NL, Louie A, Miller MH, Liu W, Deziel MR, Tam VH (2006). Quinolone efflux pumps play a central role in emergence of fluoroquinolone resistance in Streptococcus pneumoniae. Antimicrob Agents Chemother.

[12] Marrer E, Schad K, Satoh AT, Page MG, Johnson MM, Piddock LJ (2006). Involvement of the putative ATP-dependent efflux proteins PatA and PatB in fluoroquinolone resistance of a multidrug-resistant mutant of Streptococcus pneumoniae. Antimicrob Agents Chemother.

[13] Patel D, Kosmidis C, Seo SM, Kaatz GW (2010). Ethidium bromide MIC screening for enhanced efflux pump gene expression or efflux activity in Staphylococcus aureus. Antimicrob Agents Chemother.

[14] Li XZ, Nikaido H (2009). Efflux-mediated drug resistance in bacteria: an update. Drugs.

[15] Li XZ, Nikaido H (2004). Efflux-mediated drug resistance in bacteria. Drugs.

[16] Seo YS, Srinivasan U, Oh KY, Shin JH, Chae JD, Kim MY (2010). Changing molecular epidemiology of group B Streptococcus in Korea. J Korean Med Sci.

[17] European Committee on Antimicrobial Susceptibility Testing (EUCAST) Antimicrobial wild type distributions of microorganisms, 2009 and 2011 [cited 2014 Nov 18]. http://www.eucast.org/mic_.

[18] Kaatz GW, Seo SM, O’Brien L, Wahiduzzaman M, Foster TJ (2000). Evidence for the existence of a multidrug efflux transporter distinct from NorA in Staphylococcus aureus. Antimicrob Agents Chemother.

[19] Robertson GT, Doyle TB, Lynch AS (2005). Use of an efflux-deficient streptococcus pneumoniae strain panel to identify ABC-class multidrug transporters involved in intrinsic resistance to antimicrobial agents. Antimicrob Agents Chemother.

[20] Schwalbe R, Steele-Moore L, Goodwin AC (2007). Antimicrobial susceptibility testing protocols.

[21] Jones HE, Brenwald NP, Owen KA, Gill MJ (2003). A multidrug efflux phenotype mutant of Streptococcus pyogenes. J Antimicrob Chemother.

[22] Clancy J, Dib-Hajj F, Petitpas JW, Yuan W (1997). Cloning and characterization of a novel macrolide efflux gene, mreA, from Streptococcus agalactiae. Antimicrob Agents Chemother.

[23] Cai Y, Kong F, Gilbert GL (2007). Three new macrolide efflux (mef) gene variants in Streptococcus agalactiae. J Clin Microbiol.

[24] Brown MG, Mitchell EH, Balkwill DL (2008). Tet 42, a novel tetracycline resistance determinant isolated from deep terrestrial subsurface bacteria. Antimicrob Agents Chemother.

[25] Neyfakh AA, Bidnenko VE, Chen LB (1991). Efflux-mediated multidrug resistance in Bacillus subtilis: similarities and dissimilarities with the mammalian system. Proc Natl Acad Sci U S A.

[26] Brenwald NP, Gill MJ, Wise R (1998). Prevalence of a putative efflux mechanism among fluoroquinolone-resistant clinical isolates of Streptococcus pneumoniae. Antimicrob Agents Chemother.

[27] Yoshida H, Bogaki M, Nakamura S, Ubukata K, Konno M (1990). Nucleotide sequence and characterization of the Staphylococcus aureus norA gene, which confers resistance to quinolones. J Bacteriol.

[28] Iraurgui P, Torres MJ, Aznar J (2012). Molecular epidemiology of fluoroquinolone resistance in invasive clinical isolates of Streptococcus pneumoniae in Seville. Enferm Infecc Microbiol Clin.

[29] Piddock LJ, Johnson MM, Simjee S, Pumbwe L (2002). Expression of efflux pump gene pmrA in fluoroquinolone-resistant and -susceptible clinical isolates of Streptococcus pneumoniae. Antimicrob Agents Chemother.

